# Revision of the Japanese species of *Epicephala* Meyrick with descriptions of seven new species (Lepidoptera, Gracillariidae)

**DOI:** 10.3897/zookeys.568.6721

**Published:** 2016-02-23

**Authors:** Atsushi Kawakita, Makoto Kato

**Affiliations:** 1 Center for Ecological Research, Kyoto University, 2-509-3 Hirano, Otsu, Shiga 520-2113, Japan; 2 Graduate School of Human and Environmental Studies, Kyoto University, Yoshida-Nihonmatsu-cho, Sakyo 606-8501, Japan

**Keywords:** Active pollination behavior, *Breynia*, DNA barcode, *Glochidion*, Gracillariidae, Japan, obligate pollination mutualism, *Phyllanthus*

## Abstract

*Epicephala* moths are involved in obligate mutualisms with their Phyllanthaceae hosts, in which the female moths assure pollination and, in return, their progeny develop by consuming the seeds. Ecological, molecular and geographical data suggest that the genus includes several hundred species, but the majority remains to be formally described. Here we revise the Japanese species of *Epicephala* Meyrick, 1880. In addition to two previously named species, seven species are newly described: *Epicephala anthophilia* **sp. n.**, *Epicephala lanceolatella* **sp. n.**, *Epicephala perplexa* **sp. n.**, *Epicephala obovatella* **sp. n.**, *Epicephala corruptrix* **sp. n.**, *Epicephala parasitica* **sp. n.** and *Epicephala nudilingua* **sp. n.** The first four are species involved in obligate pollination mutualism, while the fifth is a pollinating seed parasite and the last two are derived non-pollinating seed parasites of herbaceous *Phyllanthus*. Each of the nine Japanese *Epicephela* species is specialized to a single plant species in the genera *Glochidion*, *Breynia* or *Phyllanthus*, except for *Epicephala obovatella* and *Epicephala corruptrix* that each utilizes two closely related *Glochidion* species. Considerable variations are found in pollination and oviposition behaviors among species, which are reflected in their proboscis and ovipositor morphologies, respectively. Molecular phylogeny indicated that there have been repeated transitions in oviposition mode during the diversification of *Epicephala*, which were accompanied by changes in ovipositor morphology, as suggested by a correlation analysis. Keys to species are provided.

## Introduction

The genus *Epicephala* Meyrick, 1880 (Gracillariidae) has recently become an important taxon in ecology and evolutionary biology because of their mutualisms with plants in the genera *Glochidion*, *Breynia* and *Phyllanthus* (Phyllanthaceae) (Kato et al. 2003; Kawakita and Kato 2009; Kawakita 2010). The females of *Epicephala* possess specialized sensilla-bearing proboscises and use them to actively collect, transport and deposit pollen on host flowers to ensure food for their seed-feeding larvae (Kato et al. 2003; Kawakita and Kato 2006; Kawakita et al. 2015). *Epicephala* moths are the only documented pollinators for many of these hosts, making this interaction an obligate mutualism. A number of *Epicephala* species have secondarily lost the pollination behavior and became parasitic (Kawakita and Kato 2009; Kawakita et al. 2015), which is often accompanied by the loss of the specialized sensilla on the proboscis (Kawakita et al. 2015).

*Glochidion*, *Breynia* and *Phyllanthus* belong to the well-defined tribe Phyllantheae, which contains over 1,200 species globally (Hoffmann et al. 2006). Of these, ca. 500 species are thought to depend exclusively on *Epicephala* for pollination (Kawakita and Kato 2009). Because there is high level of species-specificity between the plants and the moths (Kawakita and Kato 2006; Kawakita et al. 2010), a comparable number of *Epicephala* species likely exist; however, the genus currently consists of only 53 named species (De Prins and De Prins 2005, 2014; Li and Yang 2015; Li et al. 2015). There is clearly a need to rapidly advance the taxonomy of the genus, especially because most published ecological and evolutionary studies on this mutualism treated *Epicephala* with arbitrary and variable names (Kawakita and Kato 2004a,b, 2009; Kawakita et al. 2004, 2010, 2015; Okamoto et al. 2007; Hembry et al. 2012, 2013; Goto et al. 2010; Svensson et al. 2010), making comparisons among studies problematic (but see Hu et al. 2011; Zang et al. 2012a; Li and Yang 2015; Li et al. 2015 for recent taxonomic advancement of Chinese *Epicephala*).

Aside from the remarkable pollination behavior, *Epicephala* is noteworthy among other genera of Gracillariidae in being seed parasitic and having a well-developed ovipositor (Kato et al. 2003). Gracillariid moths are predominantly leaf miners; the seed-feeding habit is otherwise only known in *Conopomorpha* Meyrick, 1885, which contains species that attack seeds of tropical fruit trees such as lychee and longan (both Sapindaceae) and cacao (Malvaceae). The recently described *Conopomorpha flueggella* Li, 2011, which feeds on the seeds of *Flueggea suffruticosa* (Phyllanthaceae) (Hu et al. 2011), is distantly related to other members of *Conopomorpha* but closely related to *Epicephala* (Kawakita et al. 2010). Thus, the evolution of seed feeding and the colonization of Phyllanthaeae likely occurred in the common ancestor of *Epicephala* and *Conopomorpha flueggella*, followed by the evolution of pollination behavior, sensilla-bearing proboscis and ovipositor in *Epicephala*. Ovipositors are used by female *Epicephala* to deposit eggs internally in floral tissue (Kato et al. 2003; Kawakita et al. 2015), unlike other members of Gracillariidae that oviposit externally. Internal oviposition is secondarily lost in *Epicephala vitisidaea* Li, Wang & Zhang, 2012 and *Epicephala mirivalvata* Li, Wang & Zhang, 2012 that lay eggs in the narrow space between the tepals and ovary (Kawakita and Kato 2004; Zang et al. 2012b), although functional ovipositors are retained in both species.

The purpose of this paper is to provide descriptions of the *Epicephala* species occurring in Japan, where most published ecological studies have been conducted. The names used in published studies to refer to each species described here are also given to facilitate the interpretation of published results.

## Methods

A total of 496 adult pinned specimens were used for this study, from which 132 genital dissections were made. Descriptions focused on the adult stage because adults provide a wealth of morphological traits useful for diagnosis and because immature stages of *Epicephala* are poorly known. For genital dissections, the whole abdomen was removed and incubated for 2–4 h in 10% KOH, and residual scales and soft parts were removed in 70% ethanol. Genitalia were then stained in fuchsin acid for 30 min to 2 h, dehydrated in a series of 70−100 % ethanol and mounted in Euparal (Waldeck GmbH & Co. KG, Division Chroma, Münster, Germany) on a glass slide. To study the sensilla on the proboscis, the entire head was removed, incubated in KOH as above and mounted in Euparal on a glass slide without staining. Observation and measurements were made under an Olympus BX53F microscope at 10–40× with the aid of a micrometer scale.

Images of adults were captured using the Olympus E-330 camera mounted on Olympus SZX10 dissection microscope, and those of genitalia and mouthparts were captured using the Canon EOS Kiss X5 camera mounted on the Olympus BX53F microscope. Images were taken at various depths and subsequently stacked using the CombineZP software (www.hadleyweb.pwp.blueyonder.co.uk). All images were then edited with Adobe Photoshop CC into final figures.

To infer the phylogenetic positions of the Japanese species within *Epicephala*, we assembled published nucleotide sequence data for the cytochrome oxidase subunit 1 (COI), arginine kinase (ArgK) and elongation factor 1-alpha (EF1α) genes available in GenBank for 52 *Epicephala* species (accession numbers are provided in Suppl. material 1), and used them to reconstruct the phylogeny of the genus. Sequence data are already available for the nine *Epicephala* species treated in this study (Kawakita and Kato 2006, 2009), but they have not been analyzed together with those of other *Epicephala* for which data are available. Because a large number of *Epicephala* sequences are presently available in GenBank, only one sequence per species per locus was sampled for the analysis. The COI, ArgK and EF1α gene partitions consisted of 582 bp, 723 bp and 522 bp, respectively. Phylogeny was constructed using the maximum-likelihood method in Treefinder (Jobb 2011) with substitution models proposed by the program (GTR+G, J3+G and J2+G for COI, ArgK and EF1α, respectively). Robustness of the tree was validated by a bootstrap analysis in Treefinder with 1,000 replicates. *Conopomorpha flueggella* was included in the analysis, and *Stomphastis labyrinthica* (Meyrick, 1918), *Melanocercops ficuvorella* (Yazaki, 1926) and *Cuphodes diospyrosella* (Issiki, 1957) served as outgroups (Kawakita et al. 2010).

It was observed that *Epicephala* species that oviposit through the ovary wall possess a characteristic angular ovipositor tip, therefore it was tested whether there was a correlation between oviposition site and ovipositor morphology using the Pagel’s (1994) correlation method as implemented in the Mesquite software (Maddison and Maddison 2015). Because data on oviposition site and ovipositor morphology are also available for *Epicephala lativalvaris* Li, Wang & Zhang, 2012 (Zang et al. 2012a,b), these were included in the analysis. Oviposition site was categorized as either (1) oviposition through ovary wall or (2) oviposition in stylar tissue or external. Ovipositor morphology was categorized as either (1) angular or (2) non-angular. Using the maximum-likelihood phylogeny obtained as described above, we tested whether correlated evolution is more likely than the null hypothesis of independent evolution using Mesquite.

The level of intra- and interspecific divergences in the COI barcoding region (same as the above 582-bp fragment) was also quantified by analyzing all *Epicephala* barcode sequences presently available in GenBank. For each of the nine *Epicephala* species, the maximum pairwise distance was calculated within species and the minimal distance to other species. We also reconstructed a maximum-likelihood phylogeny in Treefinder to test for the monophyly of each species, following the method described above for the multi-gene data set. The GTR+G substitution model was chosen for the analysis.

All type materials have been deposited in the Zoological Collection of the Kyoto University Museum (KYO). Unless otherwise stated, specimens were collected by the primary author. All botanical names follow Govaerts et al. (2000).

### Keys to the Japanese species of *Epicephala*

#### Males

**Table:** 

1	Aedeagus with cornutus extending well beyond apex of aedeagus	***nudilingua***
–	Aedeagus without cornutus extending well beyond apex of aedeagus	**2**
2	Valva with a long spine 1/2 length of cucullus at base	***parasitica***
–	Valva without long spine at base	**3**
3	Sacculus with a single well developed spine at apex	***corruptix***
–	Sacculus without solo spine at apex	**4**
4	Sacculus with a row of spines running parallel to ventral margin	***perplexa***
–	Sacculus without a row of spines	**5**
5	Aedeagus with spiniform cornuti on dorsal and ventral sides	***vitisidaea***
–	Aedeagus without spiniform cornuti	**6**
6	Sacculus as long as or slightly shorter than cucullus	**7**
–	Sacculus distinctly shorter than cucullus	**8**
7	Sacculus acute apically; projection on dorsal margin hook-like	***lanceolatella***
–	Sacculus rounded apically; projection on dorsal margin round	***bipollenella***
8	Dorsal margin of sacculus with projection bearing spines	***obovatella***
–	Sacculus without spine-bearing projection	***anthophilia***

#### Females

**Table:** 

1	Seventh sternite and tergite fused laterally	***parasitica***
–	Seventh sternite connected to seventh tergite by intersegmental membrane	**2**
2	Lamella postvaginalis bilobed distally for > 1/2 of its length	**3**
–	Lamella postvaginalis not bilobed, or if bilobed, each lobe no longer than 1/2 of lamella postvaginalis itself	**5**
3	Lobe of lamella postvaginalis round, club-shaped	***obovatella***
–	Lobe of lamella postvaginalis finger-shaped	**4**
4	Lobes of lamella postvaginalis dilated laterally; signa present	***anthophilia***
–	Lobes of lamella postvaginalis straight; signa absent	***nudilingua***
5	Lamella postvaginalis heavily sclerotized and strongly curved	***perplexa***
–	Lamella postvaginalis not heavily sclerotized or strongly curved	**6**
6	Ovipositor apically bilobed and not dentate	***vitisidaea***
–	Ovipositor apically dentate and not bilobed	**7**
7	Ovipositor angular at apex	***corruptrix***
–	Ovipositor rounded at apex	**8**
8	Lamella postvaginalis > 1/2 width of seventh sternite	***lanceolatella***
–	Lamella postvaginalis < 1/2 width of seventh sternite	***bipollenella***

## Species descriptions

### Epicephala anthophilia sp. n.

Taxon classificationAnimaliaLepidopteraGracillariidae

http://zoobank.org/C379F786-7FA0-44DF-A07F-C17EE05E8A65

**Figure 1.**
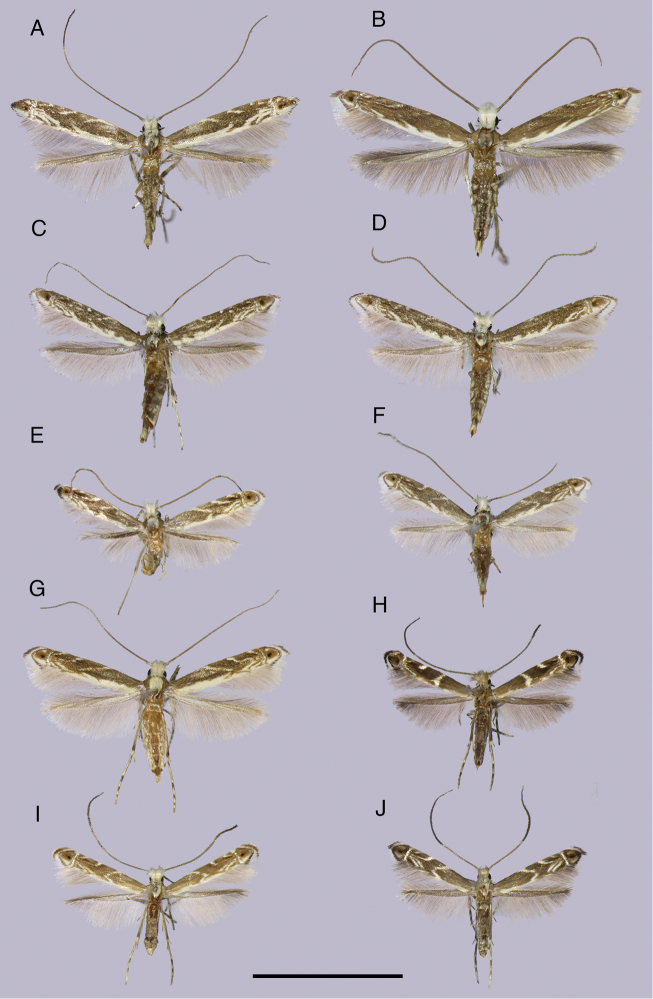
Representative specimens of the nine *Epicephala* species in Japan. Wing pattern of *Epicephala parasitica* is sexually dimorphic, so specimens of both sexes are shown for this species. **A** *Epicephala anthophilia* (Amami Island, Kagoshima, ♀, holotype) **B** *Epicephala bipollenella* (Henoko, Okinawa, ♀) **C** *Epicephala lanceolatella* (Cape Hedo, Okinawa, ♀, holotype) **D** *Epicephala perplexa* (Cape Hedo, Okinawa, ♀, holotype) **E** *Epicephala obovatella* (Tomogashima, Wakayama, ♂, paratype) **F** *Epicephala corruptrix* (Takae, Okinawa, ♀, holotype) **G** *Epicephala vitisidaea* (Yona, Okinawa, ♀) **H** *Epicephala parasitica* (Yonaguni Island, Okinawa, ♀, holotype) **I** *Epicephala parasitica* (Hateruma Island, Okinawa, ♂) **J** *Epicephala nudilingua* (Watarase-yusuichi, Tochigi, ♀, holotype). Scale bar: 5 mm.

**Figure 2.**
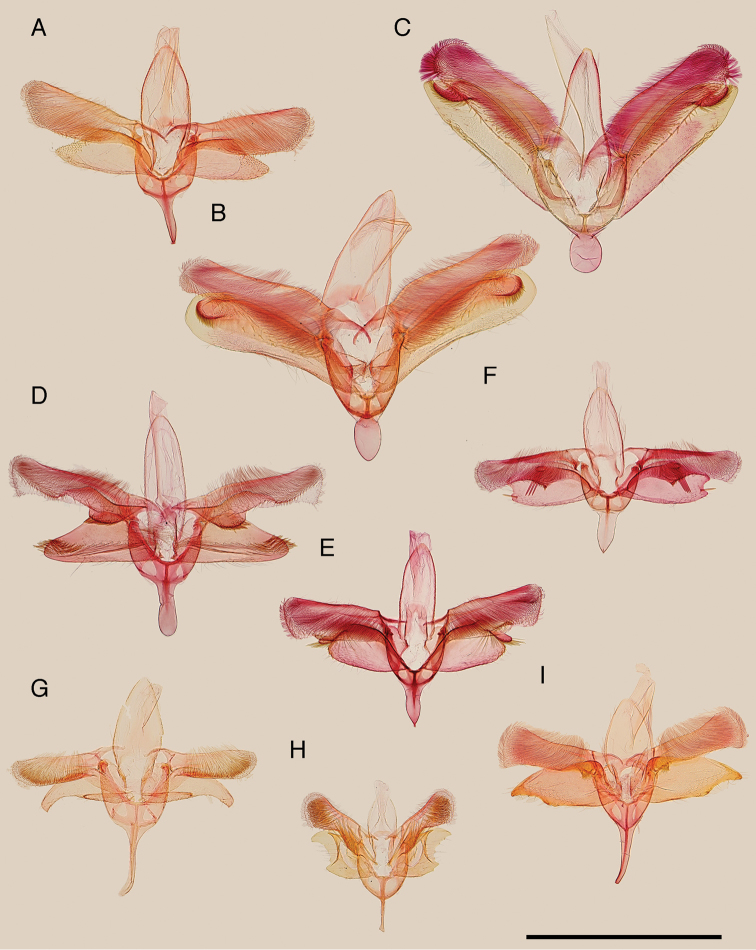
Valva of the Japanese *Epicephala* species. **A** *Epicephala anthophilia* (paratype, slide No. AK249) **B** *Epicephala bipollenella* (slide No. AK258) **C** *Epicephala lanceolatella* (non-type, slide No. AK270) **D** *Epicephala perplexa* (paratype, slide No. AK272) **E** *Epicephala obovatella* (non-type, slide No. AK245) **F** *Epicephala corruptrix* (paratype, slide No. AK260) **G** *Epicephala vitisidaea* (slide No. AK234); **H**, *Epicephala parasitica* (non-type, slide No. AK290) **I** *Epicephala nudilingua* (paratype, slide No. AK292). Scale bar: 1 mm.

**Figure 3.**
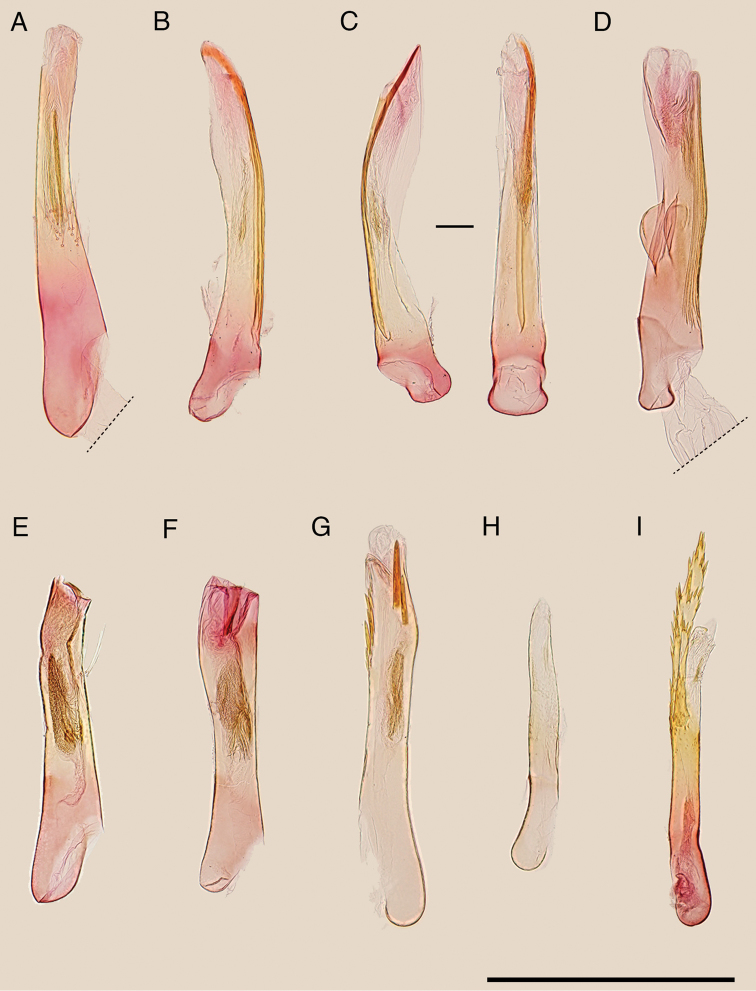
Aedeagus of the Japanese *Epicephala* species. **A** *Epicephala anthophilia* (paratype, slide No. AK249), lateral view **B** *Epicephala bipollenella* (slide No. AK258), lateral view **C** *Epicephala lanceolatella*, lateral (left; non-type, slide No. AK270) and dorsal (right; non-type, slide No. AK271) view **D** *Epicephala perplexa* (paratype, slide No. AK272), lateral view **E** *Epicephala obovatella* (non-type, slide No. AK245), lateral view **F** *Epicephala corruptrix* (paratype, slide No. AK260), lateral view **G** *Epicephala vitisidaea* (slide No. AK234), lateral view **H** *Epicephala parasitica* (non-type, slide No. AK290), lateral view **I** *Epicephala nudilingua* (paratype, slide No. AK292), ventral view. Scale bar: 0.5 mm.

**Figure 4.**
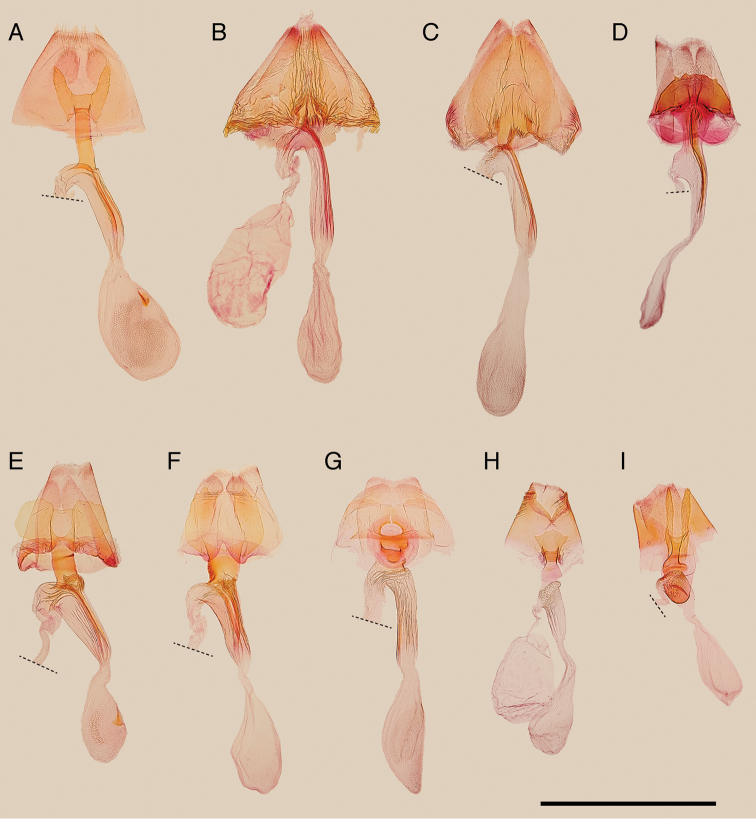
Seventh abdominal segment and corpus and ductus bursae of the Japanese *Epicephala* species. **A**, *Epicephala anthophilia* (paratype, slide No. AK250) **B** *Epicephala bipollenella* (slide No. AK281) **C** *Epicephala lanceolatella* (non-type, slide No. AK251) **D** *Epicephala perplexa* (paratype, slide No. AK253) **E** *Epicephala obovatella* (non-type, slide No. AK246) **F** *Epicephala corruptrix* (paratype, slide No. AK262) **G** *Epicephala vitisidaea* (slide No. AK239) **H** *Epicephala parasitica* (non-type, slide No. AK293) **I** *Epicephala nudilingua* (paratype, slide No. AK296). Scale bar: 1 mm.

**Figure 5.**
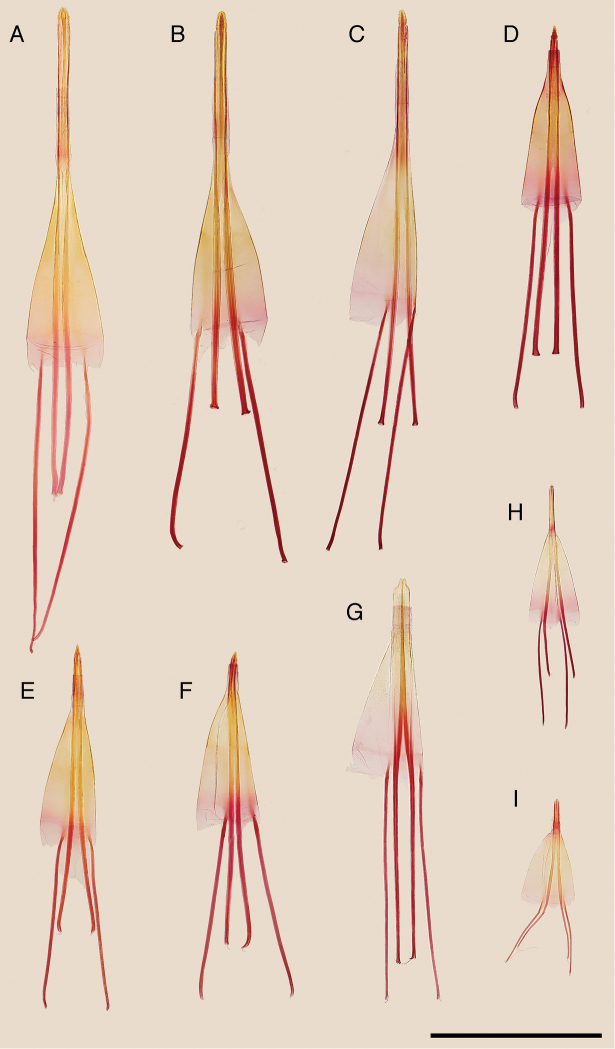
Apophyses and eighth abdominal segment of the Japanese *Epicephala* species. **A** *Epicephala anthophilia* (paratype, slide No. AK250) **B** *Epicephala bipollenella* (slide No. AK281) **C** *Epicephala lanceolatella* (non-type, slide No. AK251) **D** *Epicephala perplexa* (paratype, slide No. AK253) **E** *Epicephala obovatella* (non-type, slide No. AK246) **F** *Epicephala corruptrix* (paratype, slide No. AK262) **G** *Epicephala vitisidaea* (slide No. AK239) **H** *Epicephala parasitica* (non-type, slide No. AK239) **I** *Epicephala nudilingua* (paratype, slide No. AK296). Scale bar: 1 mm.

**Figure 6.**
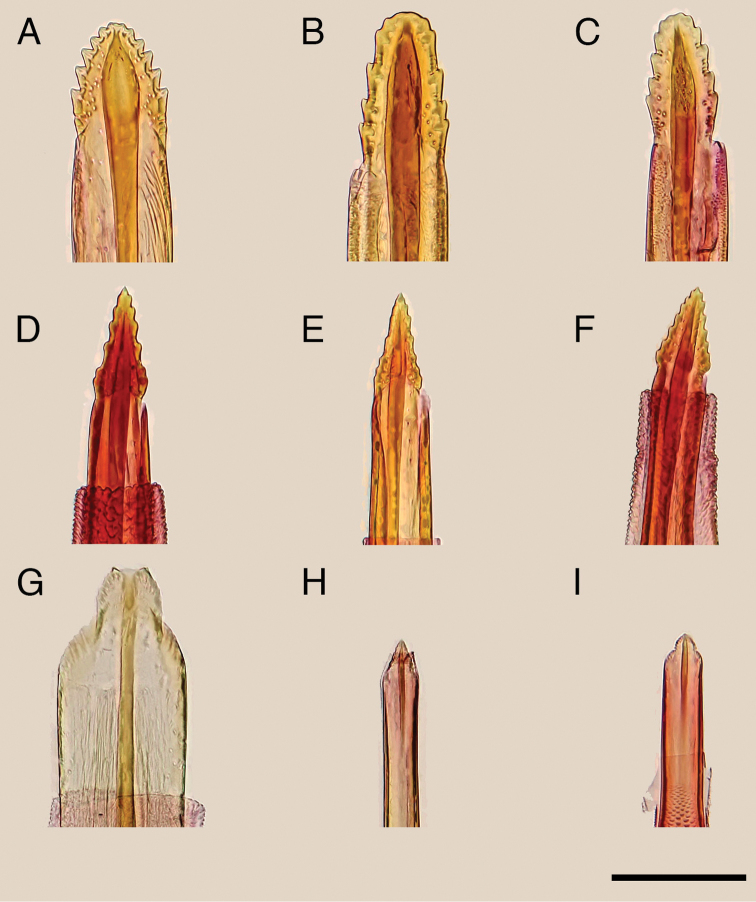
Ovipositor of the Japanese *Epicephala* species. **A**, *Epicephala anthophilia* (paratype, slide No. AK250) **B** *Epicephala bipollenella* (slide No. AK281) **C** *Epicephala lanceolatella* (non-type, slide No. AK251) **D** *Epicephala perplexa* (paratype, slide No. AK253) **E** *Epicephala obovatella* (non-type, slide No. AK246) **F** *Epicephala corruptrix* (paratype, slide No. AK262) **G** *Epicephala vitisidaea* (slide No. AK239) **H** *Epicephala parasitica* (non-type, slide No. AK239) **I** *Epicephala nudilingua* (paratype, slide No. AK296). Scale bar 0.1 mm.

**Figure 7.**
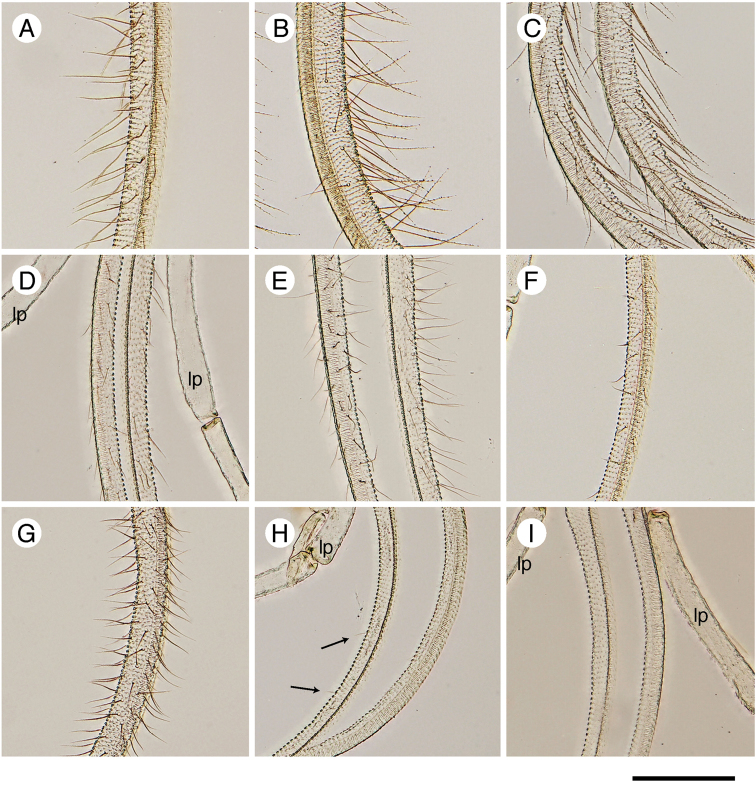
Section of the female proboscis of the Japanese *Epicephala* species. All photographs were taken from non-type specimens. **A** *Epicephala anthophilia* (slide No. AK303) **B** *Epicephala bipollenella* (slide No. AK298) **C** *Epicephala lanceolatella* (slide No. AK300) **D** *Epicephala perplexa* (slide No. AK301) **E** *Epicephala obovatella* (slide No. AK307) **F** *Epicephala corruptrix* (slide No. AK304) **G** *Epicephala vitisidaea* (slide No. AK297) **H** *Epicephala parasitica* (slide No. AK308), arrows indicate rudimentary sensilla **I** *Epicephala nudilingua* (slide No. AK309). lp, labial palp. Scale bar: 0.1 mm.

Figs 1 , 2 , 3 , 4 , 5 , 6 , 7

Epicephala sp. 1 (Kato et al. 2003); Epicephala sp. (acuminatum) (Kawakita et al. 2004); Clade 2 (Kawakita and Kato 2006); Epicephala sp. ex Glochidion acuminatum (Kawakita and Kato 2009; Kawakita et al. 2015); Epicephala sp. 2 (Glochidion acuminatum) (Kawakita et al. 2010).

#### Diagnosis.

This species is morphologically similar to *Epicephala eriocarpa* Li, Wang & Zhang, 2012 but differs from the latter in having a longer and apically acute sacculus, lamella postvaginalis with distal arms stretched outwardly, and shorter ductus bursae relative to antrum.

#### Description.

*Wingspan*: 9.2–11.0 mm.

*Head*: With numerous white scales on dorsal surface. Labial palpus with dark brown scales. Antenna brown, about 1.2× as long as forewing. Female proboscis with a large number of trichoid sensilla; sensilla 1.5× as long as width of proboscis, denser toward base.

*Thorax*: White dorsally. Forewing brown with narrow white band on dorsum from base to 2/3 of entire length; three pairs of white bands beginning at costal and dorsal margins near 1/2 to 3/4 length of wing and extending obliquely toward wing apex, terminating before reaching mid-width of wing; a narrow silver band with metallic reflection extending from costa to dorsum at 5/6 length; distal 1/6 orange-brown with black dot centrally, franked by short white spot or band near costa and dorsum; distal end fringed with narrow white band; cilia grayish brown. Hindwing brown, 0.8× length of forewing; cilia grayish brown.

*Male genitalia*: Tegumen rounded triangular. Cucullus rectangular oblong, apex rounded, inner surface covered with numerous hairs; dorsal margin longer than ventral margin. Sacculus 0.7× length of cucullus, wider than cucullus near base but abruptly tapering at mid-length, acute apically; inner surface of dorso-distal portion with minute sclerotized teeth. Vinculum U-shaped; saccus slender, 5/6 length of vinculum. Aedeagus straight; lateral surface with a pair of sclerotized longitudinal ridges near mid-length, fringed by a few spines.

*Female genitalia*: Lamella postvaginalis H-shaped, about 0.5× length of seventh abdominal segment, 4× as broad as ostium bursae; distal arms longer than basal arms and stretched outwardly. Antrum long, as long as lamella postvaginalis. Ductus bursae 1.6× length of antrum, with longitudinal parallel ridges for its entire length. Corpus bursae oval, as long as ductus bursae; signum triangular, at 1/3 from base. Apophyses posteriores about 1.5× length of apophyses anteriores. Ovipositor dentate laterally, rounded apically.

#### Material examined.

16♂, 15♀. Holotype ♀ – **JAPAN: Kagoshima Prefecture**: Amami Island, Tatsugo, Nagakumo-toge (28.447828, 129.589449), 280 m, collected on female flower of *Glochidion acuminatum* in the act of pollination and oviposition, 10.v.2015 (KYO). The specimen possesses *Glochidion* pollen on proboscis. Paratypes – same locality as holotype, 10.xii.2007, collected as larvae in fruits of *Glochidion acuminatum* and reared to adults, 6♂, 7♀ (KYO). Other specimens – **JAPAN: Kagoshima Prefecture**: Amami Island, Tatsugo, Nagakumo-toge, 4.xi.2004, 3♂; Amami Island, Asado, 8.xii.2009, 4♂, 5♀ (R. Goto); Amami Island, Setsuko, 4.xi.2004, 1♂; Amami Island, Yakugachi, 19.xii.2005, 2♂, 1♀; Amami Island, Yakugachi, 13.xii.1997, 1♀ (M. Kato).

#### DNA barcodes.

AY221964, AY221965, AY525718, DQ298944–DQ298956.

#### Known host and adult behavior.

**Figure 8.**
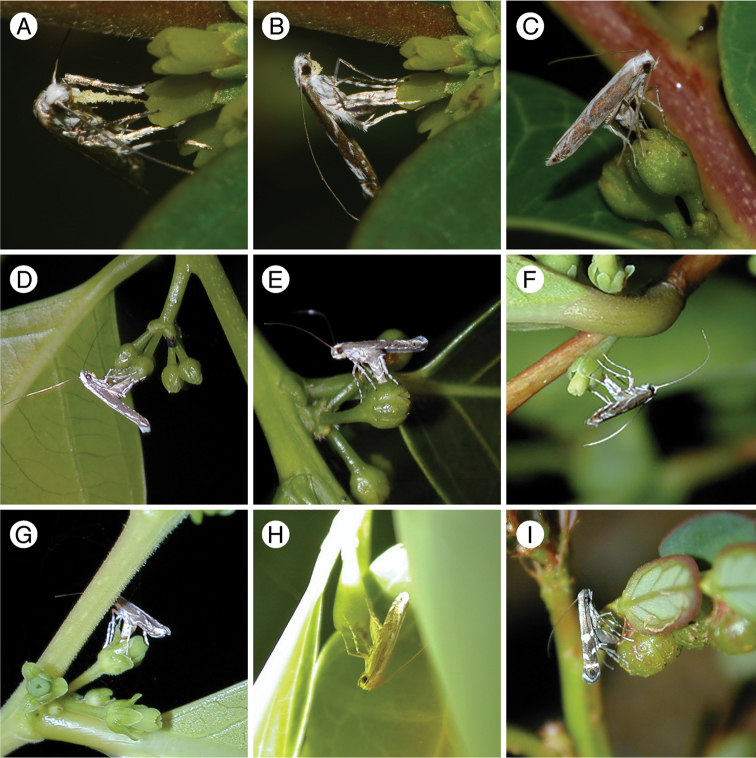
Pollination and oviposition behavior of the Japanese *Epicephala* species. **A** *Epicephala anthophilia* female actively depositing pollen on *Glochidion acuminatum* female flower **B** *Epicephala anthophilia* ovipositing through stylar pit of *Glochidion acuminatum* flower **C** *Epicephala bipollenella* ovipositing through stylar pit of *Glochidion zeylanicum* flower **D** *Epicephala lanceolatella* ovipositing through stylar pit of *Glochidion lanceolatum* flower **E** *Epicephala perplexa* ovipositing through lateral ovary wall of *Glochidion lanceolatum* flower **F** *Epicephala obovatella* ovipositing through lateral ovary wall of *Glochidion obovatum* flower **G** *Epicephala corruptrix* ovipositing through ovary wall of *Glochidion rubrum* flower **H** *Epicephala vitisidaea* ovipositing in the interspace between ovary and tepal **I** *Epicephala parasitica* ovipositing in young fruit of *Phyllanthus lepidocarpus*.

Known only from *Glochidion acuminatum*. Pollination behavior present (Fig. 8A). Oviposition from apical stylar pit, in stylar tissue (Fig. 8B). Larva feeds on seeds.

#### Distribution.

**Figure 9.**
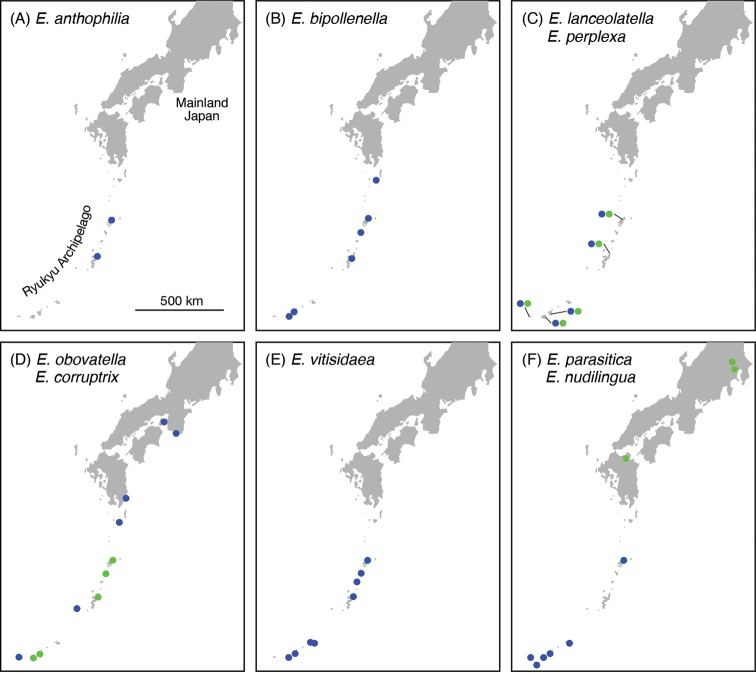
Distribution of the *Epicephala* species in Japan. **A** *Epicephala anthophilia* **B** *Epicephala bipollenella* **C** *Epicephala lanceolatella* (blue) and *Epicephala perplexa* (green) **D** *Epicephala obovatella* (blue) and *Epicephala corruptrix* (green) **E** *Epicephala vitisidaea* **F** *Epicephala parasitica* (blue) and *Epicephala nudilingua* (green). Information based on this study and Kawakita and Kato (2006).

Found in a few islands with high elevation in the Ryukyu Archipelago (Amami Island and Okinawa Island; Fig. 9A). The host plant *Glochidion acuminatum* is distributed throughout Southeast Asia from southern Japan to India, so this species is likely to be found in other parts of the host plant’s range.

#### Etymology.

The name *anthophilia* (an adjective) is derived from the Latin *antho*- (= flower) and -*philia* (= love, affection), commemorating that the flower-visiting behavior of *Epicephala* was first found in this species (Kato et al. 2003).

#### Remarks.

The flight period of this species is restricted to a 3–4 week period in May in Amami Island, corresponding to the flowering period of its host plant *Glochidion acuminatum*. The egg undergoes a prolonged dormancy in the flower for up to five months, and the larva hatches and develops as the fruit begins to mature in late October (Goto et al. 2010). The moth overwinters as pre-pupa. *Epicephala anthophilia* is presently the only known univoltine species in the genus.

### Epicephala bipollenella

Taxon classificationAnimaliaLepidopteraGracillariidae

Li, Wang & Hu, 2012

Figs 1 , 2 , 3 , 4 , 5 , 6 , 7

Epicephala sp. 2 (Kato et al. 2003); Epicephala sp. (zeylanicum) (Kawakita et al. 2004); Clade 6 (Kawakita and Kato 2006); Epicephala sp. ex Glochidion zeylanicum (Kawakita and Kato 2009); Epicephala sp. 5 (Glochidion zeylanicum) (Kawakita et al. 2010).

#### Diagnosis.

This species is distinctive among other species of *Epicephala* in that the anterior margin and midline of the seventh sternite of females have strong sclerotized wrinkles. The species is similar to *Epicephala lanceolatella* but can be distinguished from the latter by the apically rounded sacculus, stronger wrinkles on seventh sternite of females and broader lamella postvaginalis.

#### Description.

Description as in Zhang et al. (2012a), except the following.

*Head*: Female proboscis with a large number of trichoid sensilla; sensilla 1.5× as long as width of proboscis, denser toward base.

*Male genitalia*: Aedeagus slightly curved downwardly; dorsal surface with a sclerotized longitudinal ridge beginning medially at base and curving left toward apex.

#### Material examined.

64♂, 51♀. **JAPAN: Kagoshima Prefecture**: Amami Island, Akakina, 3.vii.1999, 1♂, 1♀; Amami Island, Akakina, 2.x.2002, 1♂; Amami Island, Akakina, 23.xii.2004, 2♂, 1♀; Amami Island, Taira, 24.vi.2008, 2♂, 1♀; **Okinawa Prefecture**: Okinawa Island, Henoko, 13.vi.2004, 41♂, 32♀; Okinawa Island, Henoko, 15.vi.2015, 7♂, 9♀; Okinawa Island, Taiho, 13.vi.2004, 2♂, 1♀; Okinawa Island, Higashi, 13.vi.2004, 3♂, 4♀; Ishigaki Island, Omoto, 25.ix.2005, 1♂, 1♀; Iriomote Island, Funaura, 29.ix.2004, 3♂, 1♀; Iriomote Island, Sonai, 9.ix.2008, 1♂.

#### DNA barcodes.

AY221966–AY221971, AY525733, DQ299033–DQ299039.

#### Known host and adult behavior.

Known only from *Glochidion zeylanicum*. Pollination behavior present. Oviposition from apical stylar pit, in stylar tissue (Fig. 8C). Larva feeds on seeds.

#### Distribution.

Occurs widely throughout the Ryukyu Archipelago, Japan (Fig. 9B). Known also from China (Zhang et al. 2012a).

#### Remarks.

Zhang et al. (2012a) suggest that this species pollinates two *Glochidion* species (*Glochidion zeylanicum* and *Glochidion hirsutum*, hence the name *bipollenella*). However, *Glochidion hirsutum* is a name used to refer to hairy individuals of *Glochidion zeylanicum*, which occur in low frequency in some populations. Similar co-occurrence of glabrous and pubescent individuals is common in *Glochidion* (A. Kawakita, personal observation). This species is therefore better viewed as a specialist of *Glochidion zeylanicum*, at least in populations thus far studied.

### Epicephala lanceolatella sp. n.

Taxon classificationAnimaliaLepidopteraGracillariidae

http://zoobank.org/E9626266-3849-4EB0-A73B-8A03E6CB45DC

Figs 1 , 2 , 3 , 4 , 5 , 6 , 7

Epicephala sp. (lanceolatum) (Kawakita et al. 2004); Clade 5 (Kawakita and Kato 2006); Epicephala sp. 6 (Glochidion lanceolatum) (Kawakita et al. 2010).

#### Diagnosis.

This species is very similar to *Epicephala bipollenella* but can be distinguished from the latter by the apically acute sacculus, the more curved distal appendages on sacculus and broader lamella postvaginalis.

#### Description.

*Wingspan*: 8.8–10.3 mm.

*Head*: With numerous white scales on dorsal surface. Labial palpus dark brown. Antenna brown, about 1.2× as long as forewing. Female proboscis with a large number of trichoid sensilla; sensilla 1.5× as long as width of proboscis, denser toward base.

*Thorax*: White dorsally. Forewing brown with narrow white band on dorsum from base to 2/3 of entire length; three narrow white bands beginning at dorsal margin near 1/2 to 3/4 length of wing and extending obliquely toward wing apex, terminating before reaching mid-width of wing; white spots scattered on costal half; a narrow silver band with metallic reflection extending from costa to dorsum at 5/6 length; distal 1/6 orange-brown with black dot centrally, franked by short white band near dorsum; distal end fringed with narrow white band; cilia grayish brown. Hindwing brown, 0.8× length of forewing; cilia grayish brown.

*Male genitalia*: Tegumen elongated triangular. Cucullus rounded rectangular; inner surface covered with numerous hairs. Sacculus as long and wide as cucullus, acute apically; dorsal margin rounded medially, attached with a plate possessing short spines sparsely on inner surface and terminating distally as inward hook-like projection with dense spines on dorso-ventral surface. Vinculum V-shaped; saccus oval, about 2/5 length of vinculum. Aedeagus slightly curved downwardly; dorsal surface with a sclerotized longitudinal ridge beginning medially at base and curving left toward apex.

*Female genitalia*: Lamella postvaginalis rounded triangular, bilobed at apex, as long as seventh sternite, 0.7× width of seventh sternite. Antrum short, with a pair of sclerotized parallel ridges. Ductus bursae as long as lamella postvaginalis, with longitudinal parallel ridges for its entire length. Corpus bursae elongate oval; signum absent. Apophyses posteriores 1.6× length of apophyses anteriores. Ovipositor dentate laterally, rounded apically.

#### Material examined.

36♂, 33♀. Holotype ♀ – **JAPAN: Okinawa Prefecture**: Okinawa Island, Kunigami, Cape Hedo (26.860200, 128.257979), 30 m, collected as larva in fruit of *Glochidion lanceolatum* and reared to adult, 15.vi.2015 (KYO). Paratypes – same data as holotype, 2♂ (KYO); same locality as holotype, 13.vi.2004, 11♂, 12♀ (KYO); Other specimens – **JAPAN: Kagoshima Prefecture**: Amami Island, Setsuko, 19.v.2005, 3♂; Amami Island, Naon, 24.vi.2008; **Okinawa Prefecture**: Ishigaki Island, Omoto, 30.ix.2004, 7♂, 112♀; Iriomote Island, Funaura, 5.x.2003, 3♀; Yonaguni Island, Mantabaru, 20.ix.2004, 9♂, 5♀.

#### DNA barcodes.

AY525727, DQ298957–DQ298961, DQ298965, DQ298966, DQ298968, DQ298972, DQ298973, DQ298977, DQ298981–DQ298983, DQ298986–DQ298988, DQ298990–DQ298995.

#### Known host and adult behavior.

Known only from *Glochidion lanceolatum*. Pollination behavior present. Oviposition from apical stylar pit, in stylar tissue (Fig. 8D). Larva feeds on seeds.

#### Distribution.

Ryukyu Archipelago, Japan (Amami Island, Okinawa Island, Ishigaki Island, Iriomote Island and Yonaguni Island; Fig. 9C).

#### Etymology.

The name *lanceolatella* (an adjective) derives from the species name of the host plant *Glochidion lanceolatum*.

### Epicephala perplexa sp. n.

Taxon classificationAnimaliaLepidopteraGracillariidae

http://zoobank.org/131546B9-E33D-4D3F-99AA-838AC256CF9F

Figs 1 , 2 , 3 , 4 , 5 , 6 , 7

Epicephala perplexa Clade 3 (Kawakita and Kato 2006); Epicephala sp. ex Glochidion lanceolatum (Kawakita and Kato 2009; Kawakita et al. 2015); Epicephala sp. 3 (Glochidion lanceolatum) (Kawakita et al. 2010).

#### Diagnosis.

This species is unlike any other *Epicephala* species in having outward projection on basal cucullus bearing dense spines, row of spines spanning the entire sacculus and rigidly sclerotized and ventrally curved lamella postvaginalis.

#### Description.

*Wingspan*: 8.3–10.0 mm.

*Head*: With numerous white scales on dorsal surface. Labial palpus dark brown. Antenna brown, about 1.2× as long as forewing. Female proboscis with a large number of trichoid sensilla; sensilla as long as width of proboscis, denser toward base.

*Thorax*: White dorsally. Forewing brown with narrow white band on dorsum from base to 2/3 of entire length; three narrow white bands beginning at dorsal margin near 1/2 to 3/4 length of wing and extending obliquely toward wing apex, terminating before reaching mid-width of wing; dull white spots scattered on costal half; a narrow silver band with metallic reflection extending from costa to dorsum at 5/6 length; distal 1/6 orange-brown with black dot centrally, franked by short white band near dorsum; distal end fringed with narrow white band; cilia grayish brown. Hindwing brown, 0.8× length of forewing; cilia grayish brown.

*Male genitalia*: Tegumen elongated rounded triangular. Cucullus rounded rectangular at distal half, projected outwardly at basal half with dense spines on outer surface of the projection; ventral margin of basal half folded inwardly; inner surface covered with numerous hairs. Sacculus rounded triangular, 2/3 length of cucullus, with row of long spines on ventral inner surface running parallel to ventral margin and continuing to a cluster of spines at apex. Vinculum U-shaped; saccus oblong, as long as vinculum. Aedeagus slightly curved dorsally at middle, with a pair of half-moon-shaped projections ventrally near mid-length, dorsally with parallel longitudinal ridges for entire length, dilated slightly at apex.

*Female genitalia*: Lamella postvaginalis rigid, crescent-shaped, curved ventrally, with a pair of teeth on posterior margin, as broad as and 0.5× length of seventh sternite. Antrum 1.2× length of lamella postvaginalis, with a pair of sclerotized parallel ridges continuing to ductus bursae. Ductus bursae as long as antrum, with longitudinal parallel ridges to 2/3 of its length. Corpus bursae elongate oval; signum absent. Apophyses posteriores 1.4× length of apophyses anteriores. Ovipositor dentate laterally, angular at apex.

#### Material examined.

29♂, 20♀. Holotype ♀ – **JAPAN: Okinawa Prefecture**: Okinawa Island, Kunigami, Cape Hedo (26.860200, 128.257979), 30 m, collected as larva in fruit of *Glochidion lanceolatum* and reared to adult, 15.vi.2015 (KYO). Paratypes – same data as holotype, 10♂ (KYO); same locality as holotype, 13.vi.2004, 3♂, 2♀ (KYO); Other specimens – **JAPAN: Kagoshima Prefecture**: Amami Island, Asado, 30.x.2011, 4♂, 2♀; **Okinawa Prefecture**: Ishigaki Island, Omoto, 30.ix.2004, 11♂, 10♀; Iriomote Island, Funaura, 5.x.2003, 1♂, 3♀; Yonaguni Island, Mantabaru, 20.ix.2004, 2♀.

#### DNA barcodes.

AY221977–AY221979, AY850003, DQ298962–DQ298964, DQ298967, DQ298969–DQ298971, DQ298974–DQ298976, DQ298978–DQ298980, DQ298984, DQ298985, DQ298989, DQ298996–DQ298998.

#### Known host and adult behavior.

Known only from *Glochidion lanceolatum*. Pollination behavior present. Oviposition through ovary wall, in space between the wall and ovule (Fig. 8E). Larva feeds on seeds.

#### Distribution.

Ryukyu Archipelago, Japan (Amami Island, Okinawa Island, Ishigaki Island, Iriomote Island and Yonaguni Island; Fig. 9C). Co-occurs with *Epicephala lanceolatella*.

#### Etymology.

The name *perplexa* is the female form of the Latin adjective *perplexus* (= cryptic), because this species remained hidden until a detailed study on species specificity was performed (Kawakita and Kato 2006).

#### Remarks.

**Figure 10.**
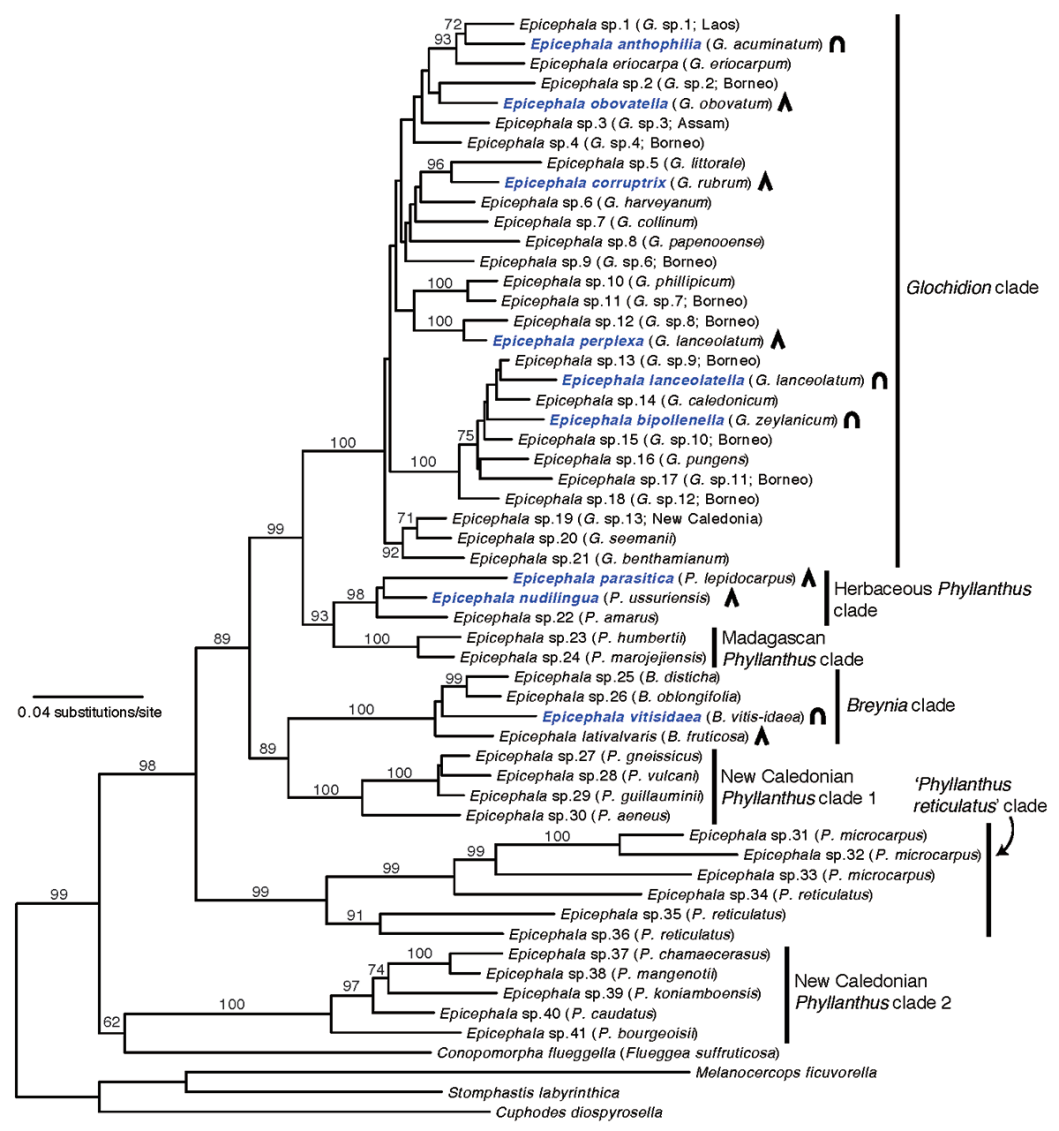
Maximum-likelihood phylogeny of *Epicephala* species based on sequences of the COI, ArgK and EF1α genes. Numbers above nodes are maximum-likelihood bootstrap support values based on 1,000 replications. The Japanese *Epicephala* species are marked in blue. Symbols right to species names donate ovipositor morphology: inverted U-shape, rounded apically; inverted V-shape, acute apically.

This species occurs in full sympatry with *Epicephala lanceolatella*. The two species may even emerge from the same single fruit. Known ecological difference between the two species is limited to the oviposition behavior, but the difference in the level of sensilla development on the proboscis (Fig. 7) may indicate that this species delivers less pollination benefit than *Epicephala lanceolatella*. Whereas the close relatives of *Epicephala lanceolatella* use plants having close affinity to *Glochidion lanceolatum*, *Epicephala perplexa* is distantly related to these *Epicephala* species (Fig. 10). Thus, the original pollinator of *Glochidion laceolatum* has likely been *Epicephala lanceolatella*, and *Epicephala perplexa* has shifted onto *Glochidion laceolatum* more recently.

### Epicephala obovatella sp. n.

Taxon classificationAnimaliaLepidopteraGracillariidae

http://zoobank.org/EB79ACCF-FDF0-47C3-8D3F-C23DBFFE6F8A

Figs 1 , 2 , 3 , 4 , 5 , 6 , 7

Epicephala sp. 3 (Kato et al. 2003); Epicephala sp. (obovatum) (Kawakita et al. 2004); Epicephala sp. (rubrum) (Kawakita et al. 2004); Clade 1 (Kawakita and Kato 2006); Epicephala sp. ex Glochidion obovatum (Kawakita and Kato 2009; Kawakita et al. 2015); Epicephala sp. 1 (Glochidion obovatum) (Kawakita et al. 2010).

#### Diagnosis.

This species is similar to *Epicephala camurella* Li, 2015 in having dented cucullus, distal projection on sacculus with dense spines, bilobed lamella postvaginalis, smooth antrum and triangular signa. However, the former clearly differs from the latter in distal projection on sacculus being finger-shaped and each lobe on lamella postvaginalis being club-shaped.

#### Description.

*Wingspan*: 7.5–11.0 mm.

*Head*: With numerous white scales on dorsal surface. Labial palpus dark brown. Antenna brown, about 1.2× as long as forewing. Female proboscis with a large number of trichoid sensilla; sensilla 1.5× as long as width of proboscis, denser toward base.

*Thorax*: White dorsally. Forewing brown with narrow white band on dorsum from base to 2/3 of entire length; three pairs of narrow white bands beginning at costal and dorsal margin near 1/2 to 3/4 length of wing and extending obliquely toward wing apex, terminating before reaching mid-width of wing; a narrow silver band with metallic reflection extending from costa to dorsum at 5/6 length; distal 1/6 orange-brown with black dot centrally, franked by short white band near dorsum; distal end fringed with narrow white band; cilia grayish brown. Hindwing brown, 0.8× length of forewing; cilia grayish brown.

*Male genitalia*: Tegumen rectangular, acute at apex. Cucullus rounded rectangular, dented at ventral margin at 3/4 of its length; inner surface covered with numerous hairs. Sacculus rounded trapezoid, 0.6× length of cucullus; dorsal margin attached with a narrow plate terminating distally as an inward finger-like projection with a row of spines dorso-ventrally. Vinculum U-shaped; saccus oblong, acute at apex, 0.8× length of vinculum. Aedeagus straight; cornutus absent.

*Female genitalia*: Lamella postvaginalis strongly bilobed; each lobe club-like, dilated outward, about 0.5× length and width as seventh sternite. Antrum broad, 0.2× width of seventh sternite, as long as lamella postvaginalis, smooth on surface. Ductus bursae 1.8× length of lamella postvaginalis, with bundle of longitudinal parallel ridges for its entire length. Corpus bursae oval, as long as buctus bursae; signum triangular, located medially. Apophyses posteriores 1.5× length of apophyses anteriores. Ovipositor dentate laterally, angular at apex.

#### Material examined.

6♂, 6♀. Holotype ♀ – **JAPAN: Wakayama Prefecture**: Wakayama, Tomogashima (34.280678, 135.000482), 12 m, collected as larva in fruit of *Glochidion obovatum* and reared to adult, 13.viii.2009 (KYO). Paratypes – same data as holotype, 1♂, 1♀ (KYO). Other specimens – **JAPAN: Wakayama Prefecture**: Wakayama, Tomogashima, 10.vii.2003, 1♂; **Miyazaki Prefecture**: Kushima, Cape Toi, 30.x.1999, 1♂, 3♀ (M. Kato); Kushima, Cape Toi, 9.xi.2001, 1♂; **Kagoshima Prefecture**: Yaku Island, Nagata, 11.xi.2001, 1♂; **Okinawa Prefecture**: Kume Island, Ueshiro, 9.viii.2004, 1♂, 1♀.

#### DNA barcodes.

AY221972–AY221976, AY525731, AY525728, DQ299001–DQ299005, DQ299008–DQ299014, DQ299019–DQ299021, DQ299023, DQ299028–DQ299032.

#### Known host and adult behavior.

*Glochidion obovatum* (mainland Japan, Yaku Island and Kume Island) and *Glochidion rubrum* (Yonaguni Island and Taiwan), which are parapatric sister species. Pollination behavior present. The egg is laid through the ovary wall between the wall and ovule (Fig. 8F). Larva feeds on seeds.

#### Distribution.

Occurs throughout the warm temperate to subtropical regions of Japan (Fig. 9D). Recorded also from Taiwan.

#### Etymology.

The name *obovatella* (an adjective) derives from the species name of the primary host plant *Glochidion obovatum*.

#### Remarks.

The Taiwanese population of this species is genetically divergent from the Japanese population (>4% divergence in COI; Table 1 and Suppl. material 2). However, they are morphologically indistinguishable (Kawakita and Kato 2006), so we tentatively consider the Taiwanese population as *Epicephala obovatella*.

**Table 1.** Maximum pairwise intraspecific and minimum interspecific divergences in the COI barcoding region for the nine Japanese *Epicephala* species.

Species	Number of DNA barcodes available in database	Maximum divergence within species (%)	Minimum divergence from other species (%)
*Epicephala anthophilia*	16	0.34	4.30
*Epicephala bipollenella*	14	0	5.01
*Epicephala lanceolatella*	24	0.17	4.66
*Epicephala perplexa*	23	0.52	4.61
*Epicephala obovatella*	28	4.12	4.30
*Epicephala corruptrix*	13	0.52	4.12
*Epicephala vitisidaea*	1	—	6.19
*Epicephala parasitica*	1	—	6.53
*Epicephala nudilingua*	1	—	5.33

### Epicephala corruptrix sp. n.

Taxon classificationAnimaliaLepidopteraGracillariidae

http://zoobank.org/4C11534C-7511-44AA-9F94-30951F9CA0A3

Figs 1 , 2 , 3 , 4 , 5 , 6 , 7

Epicephala corruptrix Clade 4 (Kawakita and Kato 2006); Epicephala sp. ex Glochidion rubrum (Kawakita and Kato 2009; Kawakita et al. 2015); Epicephala sp. 4 (Glochidion rubrum) (Kawakita et al. 2010).

#### Diagnosis.

The male genitalia of this species are distinctive and have no parallel in other known species; sacculus possesses a thick spine on apex and a cluster of long spines on dorsal projection. The large, round lamella postvaginalis also distinguishes this species from other known *Epicephala*.

#### Description.

*Wingspan*: 7.2–8.8 mm.

*Head*: With numerous white scales on dorsal surface. Labial palpus dark brown. Antenna brown, about 1.2× as long as forewing. Female proboscis with a large number of trichoid sensilla; sensilla 1.5× as long as width of proboscis, denser toward base.

*Thorax*: White dorsally. Forewing brown with narrow white band on dorsum from base to 2/3 of entire length; two pairs of narrow white bands beginning at costal and dorsal margin near 1/2 to 3/4 length of wing and extending obliquely toward wing apex, terminating before reaching mid-width of wing; dorso-distal band accompanied by another parallel band of same size on distal position; a narrow silver band with metallic reflection extending from costa to dorsum at 5/6 length; distal 1/6 orange-brown with black dot centrally, franked by short white band near dorsum; distal end fringed with narrow white band; cilia grayish brown. Hindwing brown, 0.8× length of forewing; cilia grayish brown.

*Male genitalia*: Tegumen oblong, acute at apex. Cucullus rectangular oblong; ventral margin with acute tip at mid-length terminating with short spine; inner surface covered with numerous hairs. Sacculus ovoid; apex sharply concave, with a long spine on ventral half; basal part of dorsal margin attached with a narrow plate terminating distally as an inward, finger-like projection with 3 or 4 long spines directing dorso-ventrally. Vinculum U-shaped; saccus broad, 0.3× width of vinculum, as long as vinculum, oblong and acute at apex. Aedeagus straight, slightly dilated toward apex; cornutus absent.

*Female genitalia*: Lamella postvaginalis long and broad, 0.8× width and length of seventh sternite, coarsely dentate at distal end. Antrum broad, 0.4× width and length of lamella postvaginalis, smooth on surface. Ductus bursae as long as seventh sternite, with cluster of longitudinal parallel ridges for its entire length. Corpus bursae oval to elongate oval, as long as buctus bursae; signum absent. Apophyses posteriores 1.5× length of apophyses anteriores. Ovipositor dentate laterally, angular at apex.

#### Material examined.

22♂, 5♀. Holotype ♀ – **JAPAN: Okinawa Prefecture**: Okinawa Island, Takae (26.652878, 128.248178), 100 m, collected as larva in galled flower of *Glochidion obovatum* and reared to adult, 5.vi.2015 (KYO). Paratypes – same data as holotype, 10♂, 2♀ (KYO). Other specimens – **JAPAN: Kagoshima Prefecture**: Amami Island, Akakina, 4.xi.2004, 5♂, 1♀; Amami Island, Kise, 15.iv.2006, 3♂, 1♀; Amami Island, Kasari, 18.v.2015, 1♂; Tokunoshima Island, Mikyo, 2.xi.2004, 1♂; Okinawa Prefecture: Iriomote Island, Ohara, 14.ii.1998, 2♂ (M. Kato).

#### DNA barcodes.

DQ298999, DQ299000, DQ299006, DQ299007, DQ299015–DQ299018, DQ299022, DQ299024–DQ299027.

#### Known host and adult behavior.

**Figure 11.**
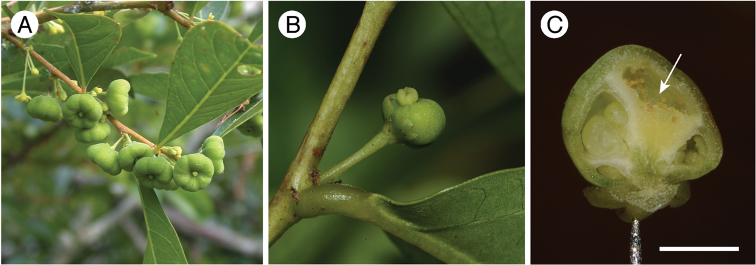
Fruits and galls produced by *Epicephala* species on *Glochidion obovatum*. **A** Fruit produced after pollination by *Epicephala obovatella* (Tomogashima, Wakayama) **B** Gall induced on female flower by *Epicephala corruptrix* (Takae, Okinawa) **C** Cross section of the gall induced by *Epicephala corruptrix*. Arrow indicates the galled locule with feeding trace of *Epicephala* larva. Note that the irregularly developed ovules of the galled locule have merged indistinguishablly to septa. Scale bar 2 mm.

*Glochidion obovatum* (Amami Island, Tokuno Island and Okinawa Island) and *Glochidion rubrum* (Ishigaki Island and Iriomote Island). The egg is laid through the ovary wall between the wall and ovule (Fig. 8G). Pollination behavior is present, but in the resulting fruit, the larva galls one of the locules, inducing abnormal development of the ovules (Fig. 11). Such fruits usually do not contain normally developed seeds (Fig. 11).

#### Distribution.

Restricted to several islands in the Ryukyu Archipelago (Amami Island, Tokuno Island, Okinawa Island, Ishigaki Island and Iriomote Island; Fig. 9D).

#### Etymology.

The species name *corruptrix* (a noun in apposition) was inherited from *Tegeticula corruptrix*, a derived parasitic species of yucca moth (Pellmyr et al. 1999). *Epicephala corruptrix* has a potential to corrupt the mutualistic relationship with its host because the species induces gall formation in pollinated flowers which then hardly produce seeds (Fig. 11).

#### Remarks.

This species shares the same host plant with *Epicephala obovatella*, but *Epicephala obovatella* has not been collected from any of the locations where *Epicephala corruptrix* occurs (Fig. 9). Because of the limited mutualistic potential of this species, reproduction of the host plants (*Glochidion obovatum and Glochidion rubrum*) is likely to be severely limited on islands where *Epicephala corruptrix* occurs, in comparison to locations where *Epicephala obovatella* is present.

### Epicephala vitisidaea

Taxon classificationAnimaliaLepidopteraGracillariidae

Li, Wang & Zhang, 2012

Figs 1 , 2 , 3 , 4 , 5 , 6 , 7

Epicephala sp. ex Breynia vitis-idaea (Kawakita and Kato 2009); Epicephala sp. 10 (Breynia) (Kawakita et al. 2010).

#### Diagnosis.

Lamella antevaginalis of this species forms a sclerotized complete circle around ostium, a character that cannot be found in any other species of *Epicephala*. Cornuti of short thick spines occurring dorsally and ventrally on distal portion of aedeagus are also distinctive of this species.

#### Description.

Description as in Zhang et al. (2012a), except the following.

*Head*: Female proboscis with a large number of trichoid sensilla; sensilla 1.5× as long as width of proboscis, denser toward base.

*Male genitalia*: Cornuti on aedeagus occurring dorsally and ventrally; dorsal cornuti consisting of 4–6 short spines, shorter than 0.7× width of aedeagus; ventral cornuti a pair of thick and long spines, longer than width of aedeagus.

#### Material examined.

50♂, 31♀. **JAPAN: Kagoshima Prefecture**: Amami Island, Setsuko, 29.ix.2002, 9♂, 1♀; Amami Island, Akakina, 15.v.2003, 5♂, 5♀; Amami Island, Kasari, 18.v.2015, 3♂; Tokuno Island, Amagi, 2.xi.2004, 1♂, 1♀; Okinoerabu Island, Uchijiro, 4.xi.2004, 1♂; **Okinawa Prefecture**: Okinawa Island, Oura, 9.ix.2002, 2♂, 3♀; Okinawa Island, Yona, 15.vi.2015, 1♂, 6♀; Miyako Island, Mt. Nobaru, 24.ix.2004, 18♂, 6♀; Irabu Island, Makiyama, 23.ix.2004, 1♂, 1♀; Ishigaki Island, Mt. Banna, 15.x.2002, 3♂, 1♀; Iriomote Island, 13.x.2002, 6♂, 7♀.

#### DNA barcodes.

FJ235380.

#### Known host and adult behavior.

Known only from *Breynia vitis-idaea*. The adult is the pollinator of the host plant (Kawakita and Kato 2004b). Eggs are placed in the interspace between the tepal and ovary (Fig. 8H), so the ovipositor does not penetrate floral tissue. Larva feeds on seeds.

#### Distribution.

In Japan occurs widely in the Ryukyu Archipelago (Fig. 9E). Known also from China (Zhang et al. 2012a).

### Epicephala parasitica sp. n.

Taxon classificationAnimaliaLepidopteraGracillariidae

http://zoobank.org/7BF0C66B-183D-4042-AE98-F059EA1EA93D

Figs 1 , 2 , 3 , 4 , 5 , 6 , 7

Epicephala sp. ex Phyllanthus lepidocarpus (Kawakita and Kato 2009; Kawakita et al. 2015); Epicephala sp. 7 (Phyllanthus) (Kawakita et al. 2010).

#### Diagnosis.

Sexually dimorphic color pattern and fused seventh sternite and tergite are thus far unknown in any species of *Epicephala*, making this species highly distinctive within the genus. Overall small size, row of thick spines on ventral margin of cucullus, long spine at cucullus base and numerous short spines on inner cucullus add to the uniqueness of this species in the genus.

#### Description.

*Wingspan*: 5.7–7.5 mm.

*Head*: Females with numerous grayish brown scales on dorsal surface; males with numerous white scales. Labial palpus dark brown to black in females, dark brown in males. Antenna dark brown in females, grayish brown in males, about 1.2× as long as forewing. Trichoid sensilla on female proboscis rudimentary, shorter than width of proboscis, less than 30 per galea.

*Thorax*: Brown dorsally in females, white in males. Forewing of females dark brown with narrow white band on dorsum from base to 1/4 of entire length, medially with narrow white band extending from costa to dorsum; a pair of narrow white bands beginning at costal and dorsal margin near 2/3 of wing and extending obliquely toward wing apex, terminating before reaching mid-width of wing; a narrow silver band with metallic reflection extending from costa to dorsum at 5/6 length; distal 1/6 brown with black dot centrally; distal end fringed with narrow white band and terminating with narrow black band; cilia dark brown. Hindwing of females dark brown, 0.8× length of forewing; cilia dark brown. Forewing of males brown with narrow white band on dorsum from base to 2/3 of entire length; three pairs of narrow white bands beginning at costal and dorsal margin near 1/2 to 3/4 length of wing and extending obliquely toward wing apex, terminating before reaching mid-width of wing; a narrow silver band with metallic reflection extending from costa to dorsum at 5/6 length; distal 1/6 orange-brown with black dot centrally, franked by short white band near dorsum; distal end fringed with narrow white band and terminating with narrow brown band; cilia grayish brown. Hindwing of males brown, 0.8× length of forewing; cilia grayish brown.

*Male genitalia*: Tegumen rounded triangular. Cucullus rectangular oblong; ventral margin medially concave; basal 1/3 of cucullus fringed with long spines on ventral margin; spines longer than width of cucullus; another distinctly long spine occurring at ventral base of cucullus, 1/2 length of cucullus; distal half of cucullus with numerous short spines on inner surface and few hairs. Sacculus broad, 2× width of cucullus, 0.7× length of cucullus, distinctly concave at apex; concave portion of apex fringed with setae; inner wall of sacculus abruptly projecting inward and curved toward dorso-caudal direction, pointed apically; ventral edge of projection fringed with setae. Vinculum U-shaped; saccus thin and rod-shaped, 0.6× length of vinculum. Aedeagus straight; cornutus absent.

*Female genitalia*: Seventh sternite completely fused to seventh tergite to form a cylindrical segment. Caudal end of seventh sternite with row of parallel latitudinal ridges. Lamella postvaginalis trapezoid, dilated toward apex, small, 0.3× width and length of seventh sternite, slightly convex and weakly dentate on caudal margin. Antrum smooth, 0.2× width and 0.5× length of seventh sternite. Ductus bursae as long as seventh sternite, with short lateral sac at base; surface of sac and franking portion of ductus bursae with numerous teeth on surface. Corpus bursae elongate oval, as long as combined antrum and ductus bursae; signum absent. Apophyses posteriores 1.7× length of apophyses anteriores. Ovipositor dentate laterally, angular at apex.

#### Material examined.

46♂, 40♀. Holotype ♀ – **JAPAN: Okinawa Prefecture**: Yonaguni Island, Sonai (24.468434, 123.002118), 50 m, collected as larvae in fruit of *Phyllanthus lepidocarpus* and reared to adult, 16.xii.2012 (KYO). Paratypes – same data as holotype, 2♂, 5♀ (KYO). Other specimens – **JAPAN: Kagoshima Prefecture**: Amami Island, Setsuko, 17.xi.2002, 2♂, 2♀; **Okinawa Prefecture**: Miyako Island, Mt. Nobaru, 24.ix.2004, 5♂, 3♀; Ishigaki Island, Omoto, 30.ix.2004, 17♂, 11♀; Iriomote Island, Funaura, 5.x.2003, 13♂, 10♀; Iriomote Island, near the mouth of Urauchi River, 29.ix.2004, 6♂, 5♀; Hateruma Island, 17.xii.2012, 1♂, 3♀.

#### DNA barcodes.

FJ235386.

#### Known host and adult behavior.

Known only from *Phyllanthus lepidocarpus*. Pollination behavior absent. Oviposition in immature fruit, through ovary wall (Fig. 8I). Larva feeds on seeds.

#### Distribution.

Widely distributed in the Ryukyu Archipelago, Japan (Fig. 9F). The host plant *Phyllanthus lepidocarpus* is a common weed along roadsides and in cultivated land. Although *Phyllanthus lepidocarpus* also occurs in mainland Japan, *Epicephala parasitica* has only been found in the Ryukyu Archipelago.

#### Etymology.

The name *parasitica* is the female form of the Latin adjective *parasiticus* (= parasitic), in reference to the parasitic nature of the species.

#### Remarks.

This and the following species (*Epicephala nudilingua*) belong to a derived clade of *Epicephala* specialized to herbaceous species of *Phyllanthus* (Fig. 10). Pollination behavior has not been observed in any of the species in this clade, so they are pure parasites that derived from a pollinating ancestor (Kawakita and Kato 2009).

### Epicephala nudilingua sp. n.

Taxon classificationAnimaliaLepidopteraGracillariidae

http://zoobank.org/462F7BC1-3195-449A-A7D7-5846ADFC724D

Figs 1 , 2 , 3 , 4 , 5 , 6 , 7

Epicephala sp. ex Phyllanthus ussuriensis (Kawakita and Kato 2009; Kawakita et al. 2015).

#### Diagnosis.

Aside from *Epicephala parasitica*, this species is smaller than any other known species of *Epicephala*. Exaggerated cornutus, spiracle on seventh tergite, bilobed lamella postvaginalis and heavily sclerotized and curved antrum, clearly distinguish this species from other known *Epicephala*.

#### Description.

Wingspan: 7.0–8.3 mm.

*Head*: With numerous gray scales on dorsal surface. Labial palpus dark brown. Antenna dark brown, about 1.2× as long as forewing. Trichoid sensilla on female proboscis absent.

*Thorax*: Grayish white dorsally. Forewing dark brown with narrow white band on dorsum from base to 1/3 of entire length; three pairs of narrow white bands beginning at costal and dorsal margin near 1/2 to 3/4 length of wing and extending obliquely toward wing apex, terminating before reaching mid-width of wing; a narrow silver band with metallic reflection extending from costa to dorsum at 5/6 length; distal 1/6 brown with black dot centrally, franked by narrow white band near dorsum; distal end fringed with narrow white band and terminating with narrow dark brown band; cilia grayish dark brown. Hindwing dark brown, 0.8× length of forewing; cilia grayish dark brown.

*Male genitalia*: Tegumen rounded triangular. Cucullus rectangular oblong, dilated at apex, covered with numerous hairs on inner surface; ventral base with small outward projection; surface of projection with numerous thin spines. Sacculus elongate triangular, acute at apex, 1.6× width of cucullus at base, 0.9× length of cucullus; distal portion of ventral margin slightly concave. Vinculum V-shaped; saccus thin and tapering, as long as vinculum. Aedeagus straight; cornutus large, emerging from 2/3 length of aedeagus and extending beyond apex of aedeagus for 0.3× length of aedeagus, 0.5× as thick as aedeagus, with thick spines sparsely on surface.

*Female genitalia*: Seventh tergite with a pair of spiracle anteriorly. Lamella postvaginalis deeply bilobed, 2× as broad as ostium bursae, as long as seventh sternite; each lobe finger-shaped, extending straight toward caudal end. Antrum heavily sclerotized, smooth on surface, abruptly curved ventrally and posteriorly to continue to ductus bursae. Ductus bursae curving abruptly anteriorly, gradually tapering to continue to corpus bursae; basal 1/3 with numerous sclerotized teeth on surface. Corpus bursae elongate oval, as long as combined antrum and ductus bursae; signum absent. Apophyses posteriores 1.7× length of apophyses anteriores. Ovipositor dentate laterally, weakly angular at apex.

#### Material examined.

19♂, 7♀. Holotype ♀ – **JAPAN: Tochigi Prefecture**: Fujioka, Watarase-yusuichi (36.226554, 139.671697), 20 m, collected as larva in fruit of *Phyllanthus ussuriensis* and reared to adult, 22.ix.2012 (KYO). Paratypes – same data as holotype, 13♂, 4♀ (KYO). Other specimens – **JAPAN: Tokyo Prefecture**: Machida, Minamiotani, 29.x.2004, 6♂, 1♀; **Oita Prefecture**: Bungo-Takada, Shinei, 8.ix.2015, 1♀ (T. Hirano).

#### DNA barcodes.

FJ235387.

#### Known host and adult behavior.

Known only from *Phyllanthus ussuriensis*. Oviposition behavior has not been observed in the wild. Floral dissection suggests that the egg is laid in young fruit through the ovary wall between the wall and ovule. Larva feeds on seeds.

#### Distribution.

Known only from three populations in Tochigi, Tokyo and Oita Prefecture, Japan (Fig. 9F). The host plant *Phyllanthus ussuriensis* is widespread in the temperate regions of Japan and other parts of East Asia, so the species is likely to be found elsewhere. The plant was common at damp habitats in flood plains before 1980s but is now uncommon and locally threatened in Japan.

#### Etymology.

The name *nudilingua* (a noun in apposition) derives from the Latin *nudus* (= naked) and *lingua* (= tongue) in reference the hairless proboscis of the female, which is a derived condition in *Epicephala*.

### Phylogenetic results

Phylogenetic analysis of the 53 *Epicephala* species resulted in a fairly resolved phylogeny (Fig. 10), consistent with previous phylogenetic analyses of *Epicephala* (Kawakita and Kato 2009; Kawakita et al. 2010, 2015; Hembry et al. 2013). *Conopomorpha flueggella* was recovered as sister to one of the two clades comprising the New Caledonian *Epicephala* species. The Japanese species belong to either one of the following three well-supported clades: the clade consisting of species associated with *Glochidion*, the clade restricted to species found on *Breynia*, and the clade containing all known species attacking herbaceous *Phyllanthus*.

Analysis of correlated evolution between oviposition site and ovipositor morphology in Mesquite indicated that the two traits exhibit greater correlation than expected under the null hypothesis of independent evolution (*P* < 0.001).

Maximum pairwise divergence in DNA barcode within species was generally low (< 1%) with the exception of *Epicephala obovatella* that exhibited moderate divergence (4.12%) between populations in Japan and Taiwan (Table 1; Kawakita and Kato 2006). Nevertheless, all *Epicephala* species for which multiple DNA barcodes were available were strongly recovered as respectively monophyletic groups in the ML phylogeny (Suppl. material 2). Minimum distances to heterospecifics were 4.12–6.53% for the nine *Epicephala* species studied (Table 1).

## Discussion

The nine species of *Epicephala* in Japan were clearly distinguishable to each other based on the morphology of both male and female genitalia, but they are usually very difficult to identify based on wing pattern. The extent of genital morphological variation of *Epicephala* is remarkable (also see Zang et al. 2012a; Li and Yang 2015; Li et al. 2015), especially when it is compared to that of other comparably large genera of Gracillariidae (e.g., *Caloptilia*) where there is very low variation in genital morphology but far greater variation in wing pattern (Kumata 1982). The morphology of the female genitalia is as diagnostic in differentiating species as that of the male genitalia, which is also uncommon in Gracillariidae. The level of interspecific genetic variation in *Epicephala* (Table 1) is not necessarily higher or even lower than those of other gracillariid genera (*Cuphodes*, 6.6–15.7%; *Diphtheroptila*, 7.3–11.6%; *Caloptilia*, 6.6–10.7%; Kawakita et al. 2010), which may indicate that genital traits in fact evolve faster in *Epicephala* than in other genera. The reason for this pattern is unknown, but there may be differences among lineages of Gracillariidae in the mechanism of reproductive isolation that can account for the observed pattern. Studies aimed at assessing the roles of genital morphology and wing pattern variation in reproductive isolation may be interesting.

The analysis of correlated evolution between oviposition site and ovipositor morphology suggested a clear linkage between these traits. Based on the phylogeny (Fig. 10), there have been repeated transitions in oviposition site during the diversification of *Epicephala*, which may indicate that shifts in oviposition site are adaptive. Because some *Glochidion* plants selectively abort flowers with heavy egg load and abortion is likely based on the extent of mechanical damage to flowers (Goto et al. 2010; personal communication of R. Goto, University of Michigan), external oviposition in *Epicephala vitisidaea* and others may have evolved to circumvent the abortion response in their host plants. By contrast, the adaptive significance of apical and lateral ovipositions is less clear. However, the observation that *Epicephala lanceolatella* and *Epicephala perplexa*, which co-occur on the same *Glochidion lanceolatum* host, display apical and lateral ovipositions, respectively, suggests that these oviposition strategies likely represent distinct niches. Detailed study on the evolution of oviposition strategy in *Epicephala* may reveal previously unknown aspect of the coevolution between the moths and their hosts.

Another finding of this study that deserves further pursuit is the galling potential of *Epicephala corruptrix* (Fig. 11). Because *Epicephala corruptrix* possesses the pollination behavior (Kawakita and Kato 2006), it is not a pure parasite. However, the benefit they confer to the plants is likely to be very small, especially when compared with *Epicephala obovatella* that uses the same *Glochidion obovatum* and *Glochidion rubrum* hosts in different locations (Fig. 9). Because our sampling is still limited, a study is needed to verify whether or not *Epicephala corruptrix* and *Epicephala obovatella* co-occur in any location and to compare their contributions to their host plant’s reproduction. *Epicephala corruptrix* will be a good model to study how shifts in the cost/benefit balance occur in mutualisms and potentially drive the collapse of the interaction.

With the seven new species described here, the genus *Epicephala* now consists of 60 described species (Li and Yang 2015; Li et al. 2015). However, the number is far lower than the few hundred species estimated from ecological, molecular and biogeographical data (Kawakita 2010). Because the present study only focused on species occurring in Japan, which is near the northern end of the distribution range of *Epicephala*, taxonomic studies encompassing broader biogeographic regions are clearly needed. For example, the molecular phylogeny of *Epicephala* (Fig. 10) suggests that there are clades confined to Madagascar or New Caledonia where none of the described *Epicephala* species occur. These regions are known for hotspots of *Phyllanthus* diversity and thus potentially have large numbers of undescribed *Epicephala* species. There is also a high diversity of *Phyllanthus* in the New World where *Epicephala* has not been recorded previously, but recent observations suggest that *Epicephala* is also widespread in the Neotropics (A. Kawakita and M. Kato, unpublished data). Accelerating the taxonomy of *Epicephala* at a global scale is therefore highly important in facilitating the ecological and evolutionary studies of this model group.

## Supplementary Material

XML Treatment for Epicephala anthophilia

XML Treatment for Epicephala bipollenella

XML Treatment for Epicephala lanceolatella

XML Treatment for Epicephala perplexa

XML Treatment for Epicephala obovatella

XML Treatment for Epicephala corruptrix

XML Treatment for Epicephala vitisidaea

XML Treatment for Epicephala parasitica

XML Treatment for Epicephala nudilingua

## Supplementary materials

Supplementary material 1 GenBank accession numbers Data type: Table Explanation note: GenBank accession numbers of the sequences used in the multi-gene phylogenetic analysis. This dataset is made available under the Open Database License (http://opendatacommons.org/licenses/odbl/1.0/). The Open Database License (ODbL) is a license agreement intended to allow users to freely share, modify, and use this Dataset while maintaining this same freedom for others, provided that the original source and author(s) are credited.Atsushi Kawakita, Makoto Kato

Supplementary material 2
COI phylogeny of *Epicephala* Data type: Figure Explanation note: Maximum-likelihood phylogeny of *Epicephala* moths based on 582 base pairs of the mitochondrial COI gene. This dataset is made available under the Open Database License (http://opendatacommons.org/licenses/odbl/1.0/). The Open Database License (ODbL) is a license agreement intended to allow users to freely share, modify, and use this Dataset while maintaining this same freedom for others, provided that the original source and author(s) are credited.Atsushi Kawakita, Makoto Kato
