# Supplementary material for: Revision of the Japanese species of Epicephala Meyrick with descriptions of seven new species (Lepidoptera, Gracillariidae)
Source: Zookeys. 2016 Feb 23;(568):87–118. doi: 10.3897/zookeys.568.6721 (PMC4829671; doi:10.3897/zookeys.568.6721)
Supplement: Supplementary material 1 — GenBank accession numbers [file zookeys-568-087-s001.pdf]

Table S1. GenBank accession numbers of the sequences used in the multi-gene phylogenetic analysis.

Species	COI	ArgK	EF-1α
Ingroup			
E. anthophilia	DQ298955	DQ298844	DQ299051
E. bipollenella	DQ299039	DQ298937	DQ299144
E. lanceolatella	DQ298960	DQ298851	DQ299058
E. perplexa	DQ298967	DQ298858	DQ299065
E. obovatella	DQ299009	DQ298903	DQ299110
E. corruptrix	DQ299027	DQ298924	DQ299131
E. vitisidaea	FJ235385	FJ235409	FJ235508
E. parasitica	FJ235386	FJ235410	FJ235509
E. nudilinguae	FJ235387	FJ235411	FJ235510
E. eriocarpa	KC912958	KC912900	KC913014
E. lativalvaris	FJ235380	FJ235404	FJ235503
E. sp. 1	KC912955	KC912897	KC913011
E. sp. 2	KC912968	KC912910	KC913024
E. sp. 3	KC912956	KC912898	KC913012
E. sp. 4	KC912962	KC912904	KC913018
E. sp. 5	KC912969	KC912911	KC913025
E. sp. 6	AY525725	AY525745	AY538758
E. sp. 7	AY525721	AY525741	AY538754
E. sp. 8	KC912922	KC912868	KC912980
E. sp. 9	KC912963	KC912905	KC913019
E. sp. 10	AY525729	AY525749	AY538762
E. sp. 11	KC912971	KC912913	KC913027
E. sp. 12	KC912967	KC912909	KC913023
E. sp. 13	N/A	KC912906	KC913020
E. sp. 14	AY525720	AY525740	AY538753
E. sp. 15	KC912965	KC912907	KC913021
E. sp. 16	AY525730	AY525750	AY538763
E. sp. 17	KC912960	KC912902	KC913016
E. sp. 18	KC912957	KC912899	KC913013
E. sp. 19	AY525735	AY525755	AY538768
E. sp. 20	AY525732	AY525752	AY538765
E. sp. 21	AY525719	AY525739	AY538752
E. sp. 22	FJ235388	FJ235412	FJ235511
E. sp. 23	FJ235379	FJ235403	FJ235502
E. sp. 24	FJ235378	FJ235402	FJ235501
E. sp. 25	FJ235381	FJ235405	FJ235504
E. sp. 26	FJ235384	FJ235408	FJ235507
E. sp. 27	FJ235376	FJ235398	FJ235497
E. sp. 28	FJ235377	FJ235400	FJ235499
E. sp. 29	AY269399	FJ235399	FJ235498
E. sp. 30	AY269393	FJ235401	FJ235500
E. sp. 31	AB978494	AB978525	AB978531
E. sp. 32	AB978496	AB978527	AB978533
E. sp. 33	AB978495	AB978526	AB978532
E. sp. 34	AB978492	AB978523	AB978529
E. sp. 35	AB978488	AB978522	AB978528
E. sp. 36	AB978493	AB978524	AB978530
E. sp. 37	AY269411	FJ235397	FJ235496
E. sp. 38	AY269397	FJ235393	FJ235492
E. sp. 39	FJ235374	FJ235394	FJ235493
E. sp. 40	FJ235375	FJ235396	FJ235495
E. sp. 41	AY269404	FJ235395	FJ235494
Outgroup			
Conopomorpha flueggella	FJ235373	FJ235392	FJ235491
Cuphodes diospyrosella	FJ235389	FJ235413	FJ235512
Stomphastis labyrinthica	FJ235390	FJ235414	FJ235513
Melanocercops ficuvorella	FJ235391	FJ235415	FJ235514
