# Supplementary material for: Revision of the Japanese species of Epicephala Meyrick with descriptions of seven new species (Lepidoptera, Gracillariidae)
Source: Zookeys. 2016 Feb 23;(568):87–118. doi: 10.3897/zookeys.568.6721 (PMC4829671; doi:10.3897/zookeys.568.6721)
Supplement: Supplementary material 2 — COI phylogeny of Epicephala [file zookeys-568-087-s002.pdf]

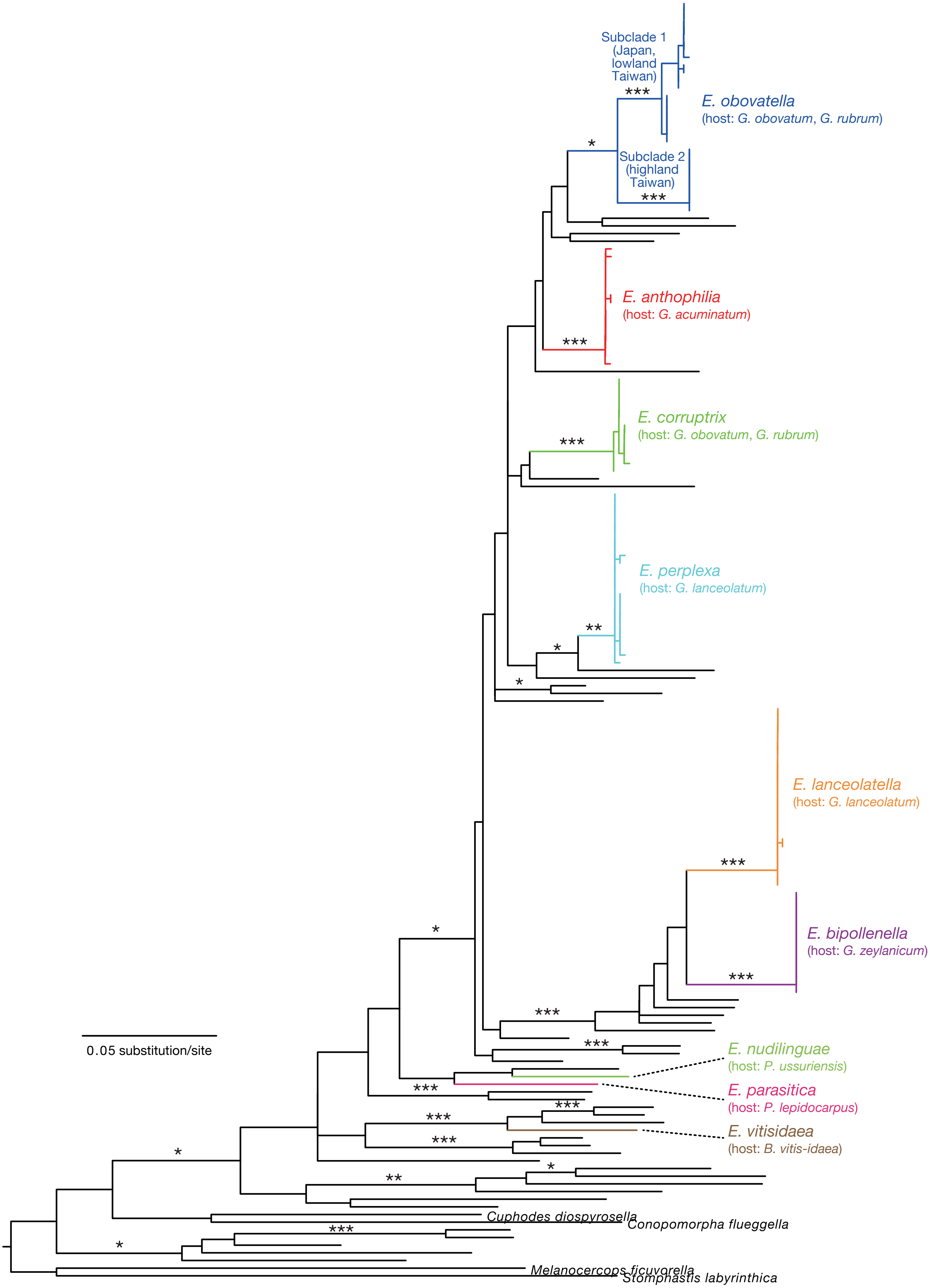

Figure S1. Maximum-likelihood phylogeny of *Epicephala* moths based on 582 base pairs of the mitochondrial COI gene. The nine Japanese species are highlighted in color. Asterisks on branches represent nodal support based on bootstrap analysis (*, >70; **, >90; ***, >95). Highland Taiwan population of *Epicephala obovatella* is genetically divergent from populations in Japan and lowland Taiwan (>4% pairwise sequence difference), but they are hardly distinguishable morphologically and thus are considered the same species.
